# Customizable landmark‐based field aperture design for automated whole‐brain radiotherapy treatment planning

**DOI:** 10.1002/acm2.13839

**Published:** 2022-11-22

**Authors:** Yao Xiao, Carlos Cardenas, Dong Joo Rhee, Tucker Netherton, Lifei Zhang, Callistus Nguyen, Raphael Douglas, Raymond Mumme, Stephen Skett, Tina Patel, Chris Trauernicht, Caroline Chung, Hannah Simonds, Ajay Aggarwal, Laurence Court

**Affiliations:** ^1^ Department of Radiation Physics The University of Texas MD Anderson Cancer Center Houston Texas USA; ^2^ Department of Radiation Oncology The University of Alabama—Birmingham Birmingham Alabama USA; ^3^ Department of Medical Physics Guy's & St Thomas NHS Foundation Trust London UK; ^4^ Division of Medical Physics Stellenbosch University and Tygerberg Academic Hospital Stellenbosch South Africa; ^5^ Department of Radiation Oncology The University of Texas MD Anderson Cancer Center Houston Texas USA; ^6^ Division of Radiation Oncology Stellenbosch University and Tygerberg Academic Hospital Stellenbosch South Africa

**Keywords:** automation, customizable field aperture, deep learning, whole‐brain radiotherapy

## Abstract

**Purpose:**

To develop and evaluate an automated whole‐brain radiotherapy (WBRT) treatment planning pipeline with a deep learning–based auto‐contouring and customizable landmark‐based field aperture design.

**Methods:**

The pipeline consisted of the following steps: (1) Auto‐contour normal structures on computed tomography scans and digitally reconstructed radiographs using deep learning techniques, (2) locate the landmark structures using the beam's‐eye‐view, (3) generate field apertures based on eight different landmark rules addressing different clinical purposes and physician preferences. Two parallel approaches for generating field apertures were developed for quality control. The performance of the generated field shapes and dose distributions were compared with the original clinical plans. The clinical acceptability of the plans was assessed by five radiation oncologists from four hospitals.

**Results:**

The performance of the generated field apertures was evaluated by the Hausdorff distance (HD) and mean surface distance (MSD) from 182 patients’ field apertures used in the clinic. The average HD and MSD for the generated field apertures were 16 ± 7 and 7 ± 3 mm for the first approach, respectively, and 17 ± 7 and 7 ± 3 mm, respectively, for the second approach. The differences regarding HD and MSD between the first and the second approaches were 1 ± 2 and 1 ± 3 mm, respectively. A clinical review of the field aperture design, conducted using 30 patients, achieved a 100% acceptance rate for both the first and second approaches, and the plan review achieved a 100% acceptance rate for the first approach and a 93% acceptance rate for the second approach. The average acceptance rate for meeting lens dosimetric recommendations was 80% (left lens) and 77% (right lens) for the first approach, and 70% (both left and right lenses) for the second approach, compared with 50% (left lens) and 53% (right lens) for the clinical plans.

**Conclusion:**

This study provided an automated pipeline with two field aperture generation approaches to automatically generate WBRT treatment plans. Both quantitative and qualitative evaluations demonstrated that our novel pipeline was comparable with the original clinical plans.

## 1 INTRODUCTION

Brain metastases are the most common intracranial malignancies in the adult population, contributing to 308,102 new cases and 251,329 deaths worldwide based on the reports in the global cancer statistics 2020.^1^ More than 40% of patients with cancer develop brain metastases.^2^ Whole‐brain radiotherapy (WBRT) treatment is a well‐established treatment for patients with brain metastases by radiologically controlling both visible tumors and invisible micrometastases.^3^

Conventional WBRT treatment planning has many challenges. First, it is time‐consuming to obtain input from physicians, physicists, and dosimetrists regarding contours and field shape setups, including multileaf collimator (MLC) blocking. After the completion of computed tomography (CT) simulation, it can take several hours to a day to obtain the input from all members of the radiotherapy team needed to start treatment.^4^ In addition, institutions vary in their clinical approaches to WBRT treatment planning. Furthermore, limited resources in low‐to‐middle‐income countries can lead to delays, especially in regard to human resources.^5^ For these reasons, automation may improve the efficiency of WBRT, and it may enable medical staff to refocus their efforts on developing more complex treatment plans. It is essential, however, for automated WBRT to be customizable for individual clinical practices.^6^, ^7^

With the development of artificial intelligence (AI) techniques, automation of the WBRT treatment planning process has greatly simplified clinical workflows and improved the quality of treatment planning.^8^ The WBRT planning process can be automated by using deep learning to predict field apertures.^9^ In Han et al.’s work, DeepLabV3+ architecture was trained to automatically define the beam apertures on lateral‐opposed fields using digitally reconstructed radiographs (DRRs).^9^ However, the approach lacks flexibility; it cannot be configured to suit local clinical practices. By providing anatomical landmarks as the rules, a configurable solution can address the limitation of lacking flexibility, which requires configurability to accommodate different clinical preferences and disease specifications. As radiation safety is critical to patients undergoing radiation therapy, quality assurance (QA) is a common way to detect potential errors or plan failures and alert people in advance. Thus, to ensure treatment planning quality, we developed a secondary approach that can equally be performed in generating radiation field apertures as the first approach. Automating the treatment planning process is part of a general trend toward full automation and will be beneficial for clinical teams to scale their efforts to treat more patients with reduced time between CT (simulation) scan and WBRT treatment.

## 2 METHODS

**FIGURE 1:**
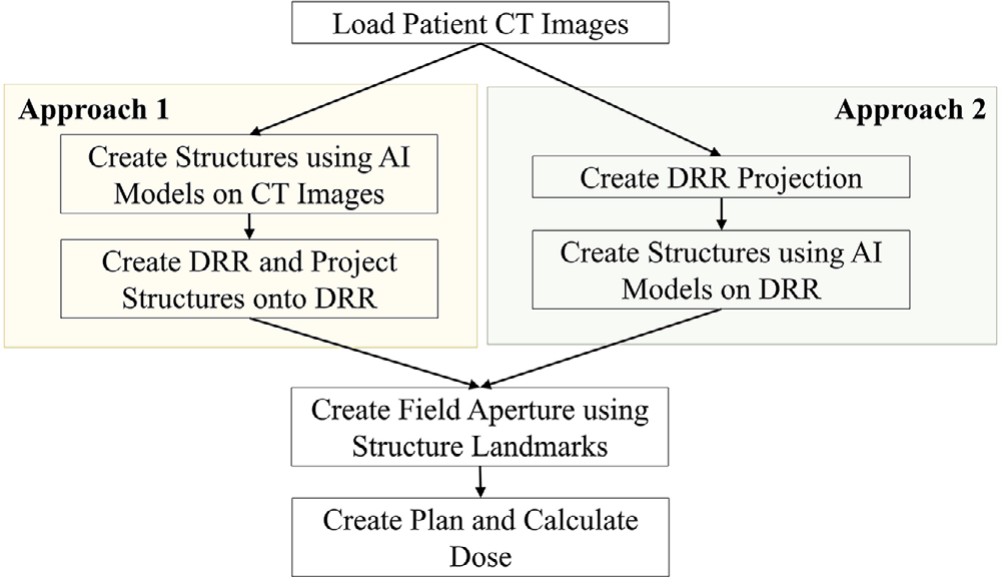
Automated whole‐brain radiotherapy (WBRT) treatment planning workflow

After loading the CT simulation images, the automated WBRT pipeline includes (1) create structures using deep learning methods, (2) create field apertures using structure landmarks, and (3) create plan and calculate dose (Figure 1). We developed two parallel automated approaches for generating field apertures. The approaches rely on anatomic landmarks (locations or points used to define the boundaries of the field apertures) in the beam's‐eye‐view but differ in how these landmarks are generated. The first approach generates landmarks by auto‐contouring various structures on the three‐dimensional (3D) CT images and then projecting them into the beam's‐eye‐view. The second approach auto‐contours the same structures directly in the beam's‐eye‐view using 2D DRRs. Two approaches were developed so that one could be used for automated planning, and one could be used to verify the first approach, similar to automated QA approaches described preciously.^10^, ^11^ In both approaches, the beam's‐eye‐view contours are used to guide aperture creation. The details of the automated plan creation can be found in Section S1.

### 2.1 Auto‐contouring

Structures for defining the anatomic landmarks (the brain, eyes, lenses, and C1 and C2 vertebral bodies) were contoured by deep learning–based auto‐contouring. For the first approach, 3D CT was used for auto‐contouring, using models previously trained and validated by our research team: The brain, eyes, and lenses were contoured using Rhee et al.’s head‐and‐neck model,^10^ and vertebral bodies (C1 and C2) were contoured using models by Netherton et al.^12^ These authors used the following convolutional neural networks‐based deep learning architectures: FCN‐8s (brain), 3D V‐Net (eyes and lens), and UNet++ (vertebral body).^10^, ^12^ For the second approach, 2D DRRs and the structures mentioned earlier were generated based on the 3D CT image set on beam's‐eye‐view (270‐degree gantry angle) using in‐house software. Deep learning models were trained using DRRs from 1263 patients who were previously treated with WBRT at our institution, including 887, 194, and 182 patients as the training, validation, and testing sets, respectively. The deep learning network was built based on fully convolutional networks with an output stride of 8 (FCN‐8s^13^) architecture for each structure, which consists of 15 convolutional layers and 5 max pooling layers (same architecture in 3D CT brain segmentation).

### 2.2 Customizable landmark‐based field aperture design

**FIGURE 2:**
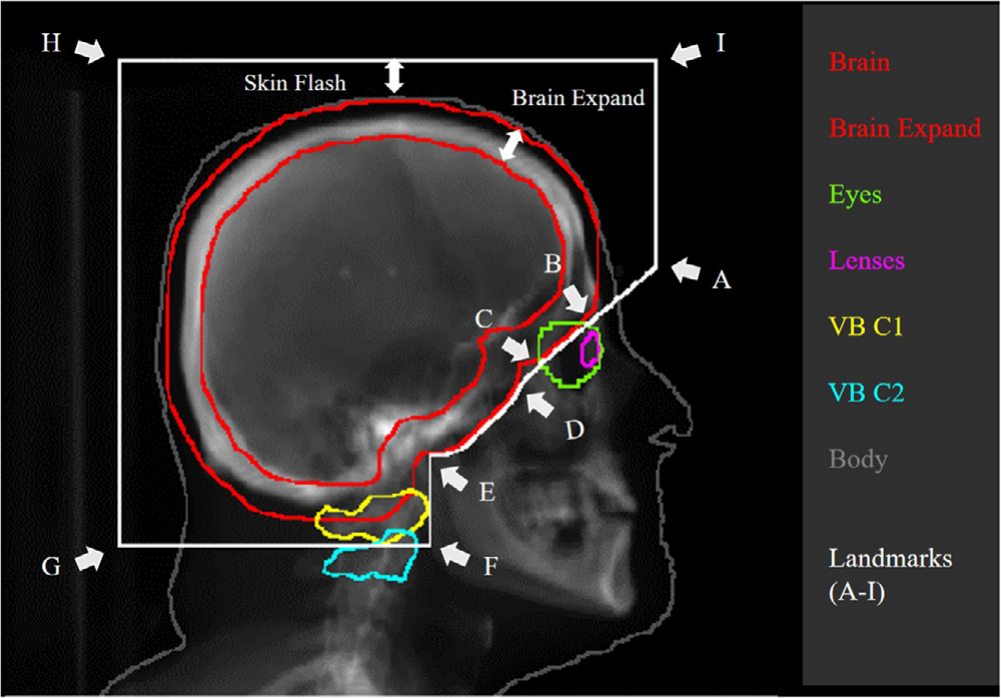
Field aperture design using different landmarks

The landmarks are the locations or points used to define the boundaries of the field apertures. In this work, landmarks were defined by the anatomic locations of structures on the beam's‐eye‐view. Based on the structures on the beam's‐eye‐view, we use bounding boxes to calculate the coordinates of the four boundaries, including left, right, top, and bottom boundaries for each structure. Then we mark the corresponded locations of the boundaries on DRR. Considering different structures have different importance to the field aperture design, we select partial boundaries to mark as landmarks. As shown in Figure 2, nine landmarks (A–I) were calculated to form the cranial (HI), caudal (FG), anterior (AI, EF), posterior, and anterior–caudal (AB, BC, CD, and DE) boundaries of the radiation field aperture.

The entire brain was considered to be the treatment target. As the eyes and lenses are sensitive to radiation, we needed to locate them and adjust the MLC to provide proper protection. Our proposed design is customizable and allows the exact relationship between the field aperture and these structures to be adjusted on the basis of local clinical requirements. The customizable options include the anterior–caudal boundary shapes, the extent of brain expansion, and the skin flash extension. Brain expansion is a morphological dilation of brain contour on the beam's‐eye‐view with a certain dilation size in mm. Skin flash extension is the distance between skin and the nearest field aperture boundary, which allows the user to extend the fluence outside the skin on the beam's‐eye‐view. In addition to these stylistic differences, the field shapes must also be adjustable based on the specifics of the patient's disease. That is, patient‐specific options, including the choice of having vertebral body C1 or C2 in the aperture and whether to include the orbitals. Different options for the visualization of the field shapes are shown in Figure 2.

### 2.3 Initial field aperture configuration selection

**FIGURE 3:**
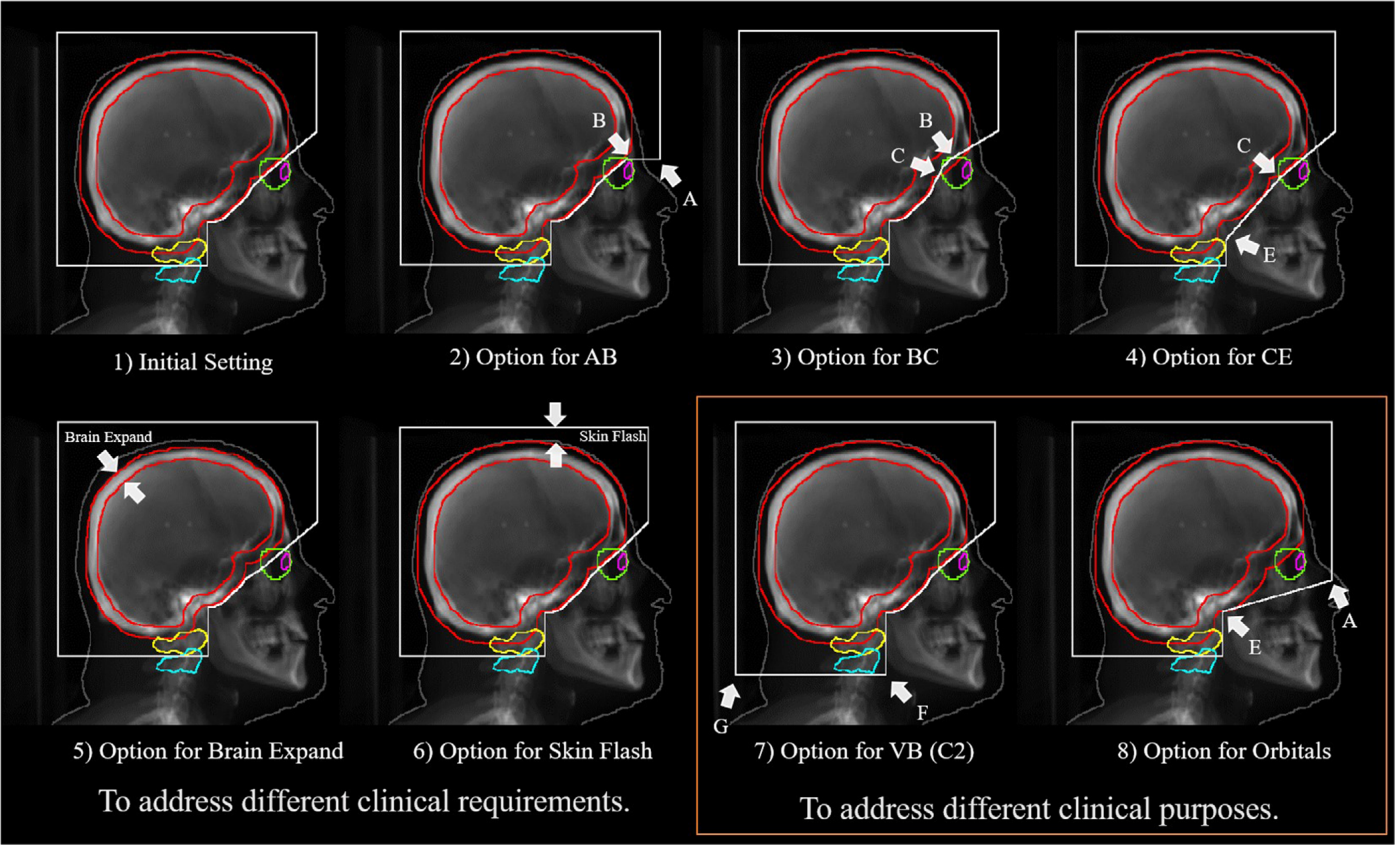
Generated field apertures based on different options. Options 1–6 are configured to address different clinical preferences, and options 7 and 8 are configured to address different clinical purposes: (1) the initial setting; (2) the line shapes for AB (horizontal or slash/diagonal‐like); (3) the option of adjusting the line BC at different distances between the eyes and the cribriform plates (close to the lenses or eyes at the cranial–posterior boundary); (4) the line shapes for CE (along the brain expand or a straight line directly connected CE); (5) different sizes for the extent of brain expansion (10, 15, or 20 mm); (6) different sizes for the extent of skin flash (10, 15, or 20 mm); (7) the selection of the vertebral bodies C1 and C2; (8) the option of treatment includes the orbitals.

The initial field aperture configuration was determined by seven radiation oncologists with a minimum of 6 years of experience and from five institutions worldwide. Evaluations were conducted using the scans of five patients. For each patient, 12 candidate field apertures (Figure S1) were generated based on different combinations of the landmark‐based options (Figure 3 options 2, 4–6). For example, candidate (a) in Figure S1 was combined using option 2 with a horizontal line, option 4 for along the brain expand, option 5 with a 15 mm brain expansion, and option 6 with a 15 mm skin flash. The reviewers ranked the top three field aperture shapes they preferred to use for each patient. A general score based on the five‐point Likert scale was given to each of the 12 candidate field shapes for each patient. The five‐point Likert scale was defined as 5—Strongly agree, which is use‐as‐is (i.e., clinically acceptable and could be used for treatment without change). Then, 4—Agree, which needs minor edits that are not necessary, with stylistic but not clinically important differences, and the current contours/plan are acceptable; and 3—Neither agree nor disagree, which requires minor edits that are necessary. Minor edits are those that the reviewer judges can be made in less time than starting from scratch or those that are expected to have minimal effect on treatment outcome. Then comes the option 2—Disagree, which requires major edits. The necessary edits are required to ensure appropriate treatment and sufficiently significant that the user would prefer to start from scratch. Finally, 1—Strongly disagree, which is unusable. The quality of the automatically generated contours or plan is so bad that they are unusable.

**FIGURE 4:**
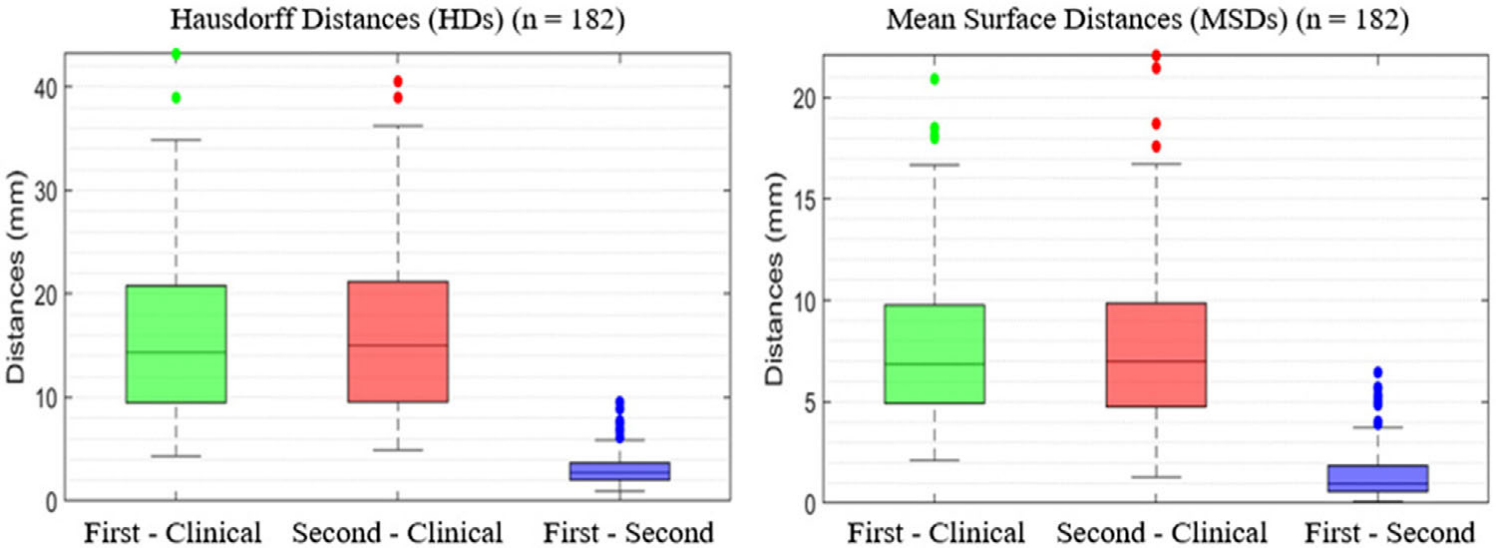
Box plots of the quantitative metrics of the field aperture design indicating the first and second approaches performed similarly

The initial field aperture configuration was then defined as the shape that received the highest rating and the highest scores. During the implementation of the field apertures based on the initial setting, clinicians’ feedback was collected each time an adjustment was made. The reviewers were asked to provide general comments about what reviewers like or dislike about the generated field apertures, such as a tighter or looser boundary at different structures.

### 2.4 Performance evaluations

We evaluated the performance of the field aperture designs and the generated plans using the initial field aperture configuration. Scans from 182 patients (refer to Section 2.1, the second approach's testing set) from our institution were included in the assessment of the quality of the generated field apertures. Further assessments, including dose coverage calculation and physician review on the generated plans, were evaluated based on 30 patients that were randomly selected from the 182 testing group. Plan generation details can be found in Section S1.

#### 2.4.1 Quantitative evaluation

The quantitative evaluation metrics for field aperture design included the Hausdorff distance (HD) and mean surface distance (MSD) that were computed to the clinical plans. Radiation doses to the structures were evaluated by calculating the maximum dose to the lenses, the mean dose to the eyes, and the brain volume receiving 95% of the prescribed dose (*V*95%).

#### 2.4.2 Qualitative Evaluation

To qualitatively assess the performance of the two approaches, physician reviews were performed on the design of the field apertures and the treatment plans. Five experienced radiation oncologists from four hospitals reviewed both the first and the second approaches using the plans generated from 30 patients. A five‐point Likert scale scores greater or equals 3 was used to determine clinical acceptability. Reviewers were asked to give a score for the field apertures and a score for the plans regarding the dose metrics, including the isodose distribution on the transverse plane, the brain dose coverages, the maximum dose to the lens, the mean dose to the eyes, and the dose–volume histogram.

## 3 RESULTS

Clinicians’ feedback was collected each time an adjustment was made during the implementation of the field apertures. The initial field aperture configuration (Figure 3) was selected based on the field apertures that received the highest scores from the reviewers (Table 1). The initial configuration included (1) the slash/diagonal‐like AB line shape, (2) the CD, DE line shape along with the brain expansion, (3) the brain expansion sets 1.5 cm, (4) the skin flash sets 1.5 cm, and (5) line BC at moderate distances between the backs of the lenses and eyes. We evaluated the deep learning model segmentation performance by calculating the Dice scores for the generated contours and the ground truths delineated by the physicians. Initial assessment of the model performance gave mean Dice similarity coefficient of organ contours segmented on DRRs regarding brain, eyes, lens, C1, and C2 were 0.97 ± 0.01, 0.88 ± 0.05, 0.55 ± 0.18, 0.89 ± 0.04, and 0.87 ± 0.07, respectively, indicating that it was adequate for calculating field apertures.

**TABLE 1:** Initial field aperture configuration review results

	Configuration	a	b	c	d	e	f	g	h	w	x	y	z
Sum	Patient 1	30	21	29	20	23	24	**33**	22	31	21	23	22
	Patient 2	28	27	**29**	26	23	25	26	24	27	25	22	24
	Patient 3	27	22	25	21	19	19	**29**	21	27	20	19	19
	Patient 4	27	22	27	20	23	23	28	22	**29**	22	23	24
	Patient 5	26	20	25	19	22	21	**27**	20	**27**	20	22	22
Average	Patient 1	4	3	4	3	3	3	**5**	3	4	3	3	3
	Patient 2	4	4	4	4	3	4	4	3	4	4	3	3
	Patient 3	4	3	4	3	3	3	4	3	4	3	3	3
	Patient 4	4	3	4	3	3	3	4	3	4	3	3	3
	Patient 5	4	3	4	3	3	3	4	3	4	3	3	3
Mode	Patient 1	4	3	**5**	2	4	4	**5**	3	**5**	3	4	3
	Patient 2	4	4	4	4	4	4	**5**	4	**5**	**5**	4	4
	Patient 3	4	3	4	4	3	3	**5**	2	**5**	2	3	3
	Patient 4	4	3	**5**	4	4	4	**5**	2	**5**	3	3	4
	Patient 5	4	3	4	3	4	4	**5**	2	**5**	2	3	4
Ranks	Mode	3	1	1	2	0	0	**1**	3	2	3	2	2
	Number of best	1	2	4	0	0	0	**17**	1	5	0	0	0

*Note*: The reviews were conducted on five patients by seven radiation oncologists. The review results regarding the sum, the average, and the mode were scores based on the five‐point Likert scale mentioned in Section 2.3. The ranks were reported using the mode of the top three configurations and the number of the ranks that were ranked as the best among all reviewers and patients (best results in bold red).

### 3.1 Quantitative evaluation

We selected a rectangle region from the anterior (around 20 mm) of the forehead to the back (around 5 mm) of the vertebral bodies for HD and MDS calculations. The average HD and MSD for the generated field apertures were 16 ± 7 and 7 ± 3 mm, respectively, for the first approach, and 17 ± 7 and 7 ± 3 mm, respectively, for the second approach (Figure 4). The differences in HD and MSD between the first and the second approaches were 1 ± 2 and 1 ± 3 mm, respectively. Although the distances between the manual and automated approaches were fairly large, the majority of distances were contributed in less critical regions, such as above eyes and behind vertebral bodies (Figure 6).

The different dose metrics compared between the clinical plans and the plans generated using the first and second approaches, including the brain *V*95%, the maximum doses to the lenses, and the mean dose to the eyes from 30 patients (Table 2). Both landmark‐based approaches achieved a 100% acceptance rate from the radiation oncologists for brain *V*95%. The average acceptance rates for meeting lens dosimetric recommendations were 80% (left lens) and 77% (right lens) for the first approach, and 70% (both left and right lenses) for the second approach, compared with 50% (left lens) and 53% (right lens) for the clinical plans, indicating that the automated plans maintained dose coverage of the brain with some reduction in the lens dose.

**TABLE 2:** Comparison of different dose metrics

Target/OAR	Plan type	Dose/volume metric	Clinical constraint	Resultant dose	Clinical dose	Resultant acceptance rate (*n* = 30) (%)	Clinical acceptance rate (*n* = 30) (%)
Brain	First approach	*V*95%	>95%	99.97 ± 0.16%	99.97 ± 0.17%	100	100
	Second approach	*V*95%	>95%	99.97 ± 0.16%		100	
Left lens	First approach	Max dose	<8 Gy	5.90 ± 2.95 Gy	11.11 ± 9.24 Gy	80	50
	Second approach	Max dose	<8 Gy	6.22 ± 3.62 Gy		70	
Right lens	First approach	Max dose	<8 Gy	6.57 ± 3.94 Gy	12.23 ± 10.11 Gy	77	53
	Second approach	Max dose	<8 Gy	6.65 ± 4.34 Gy		70	
Left eye	First approach	Mean dose	n.a.	10.34 ± 1.85 Gy	13.21 ± 7.01 Gy	n.a.	n.a.
	Second approach	Mean dose	n.a.	9.85 ± 3.01 Gy		n.a.	
Right eye	First approach	Mean dose	n.a.	10.91 ± 1.93 Gy	13.71 ± 7.03 Gy	n.a.	n.a.
	Second approach	Mean dose	n.a.	10.38 ± 2.87 Gy		n.a.	

*Note*: The results were compared between the clinical plans and the plans generated using the first and second approaches, including the brain *V*95%, the maximum doses to the lenses, and the mean dose to the eyes from 30 patients. The acceptance rate for different approaches was calculated based on the clinical constraints.

Abbreviations: n.a., not applicable; OAR, organ at risk.

### 3.2 Qualitative evaluation

**FIGURE 5:**
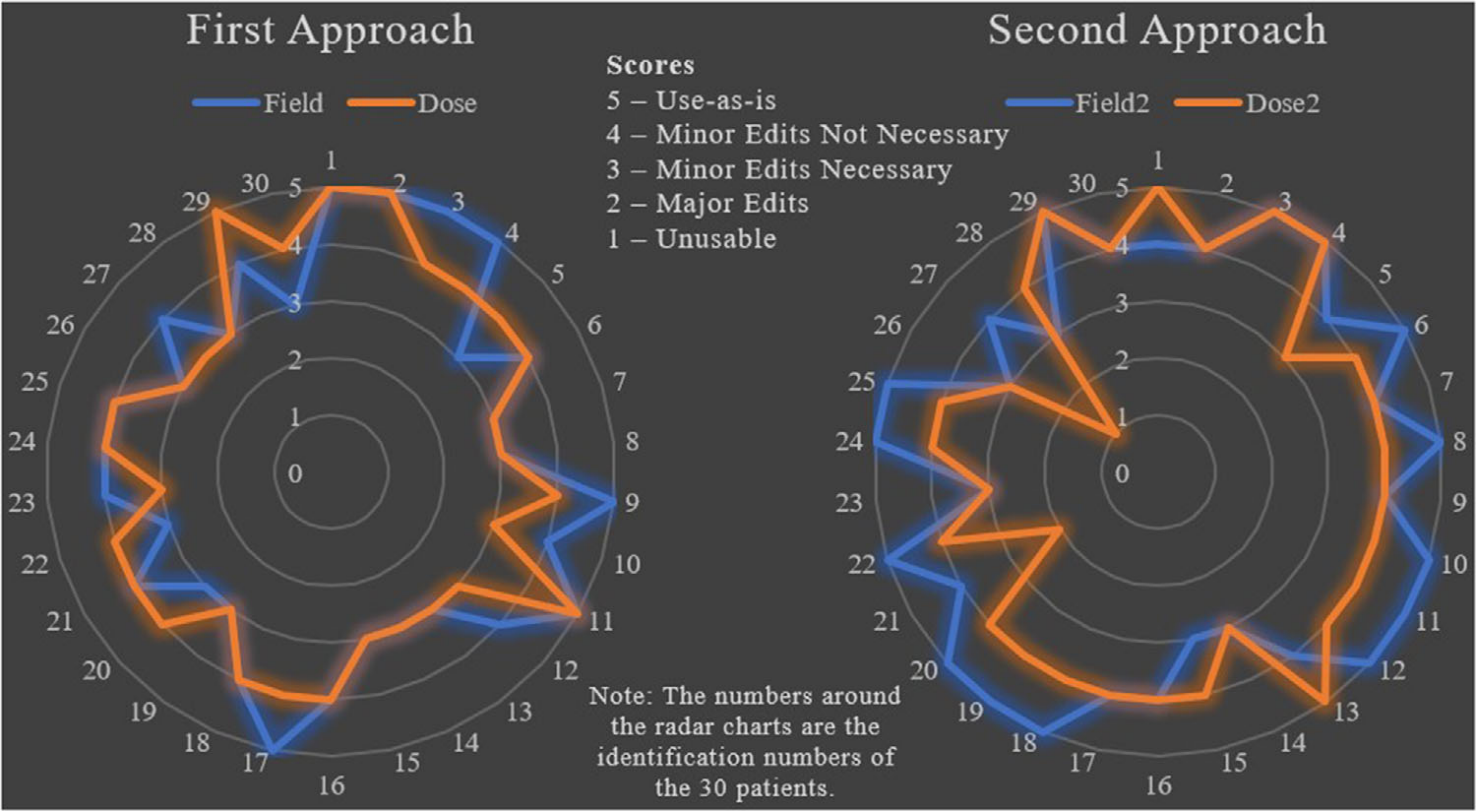
Radar plots of the clinical review results based on a five‐point Likert scale

Clinical reviews for the field aperture design and the plans were conducted on 30 patients using the five‐point Likert scale (Figure 5). The field aperture designs created using the first and second approaches both achieved a 100% acceptance rate, and the plans created using the first and second approaches achieved acceptance rates of 100% and 93%, respectively.

**FIGURE 6:**
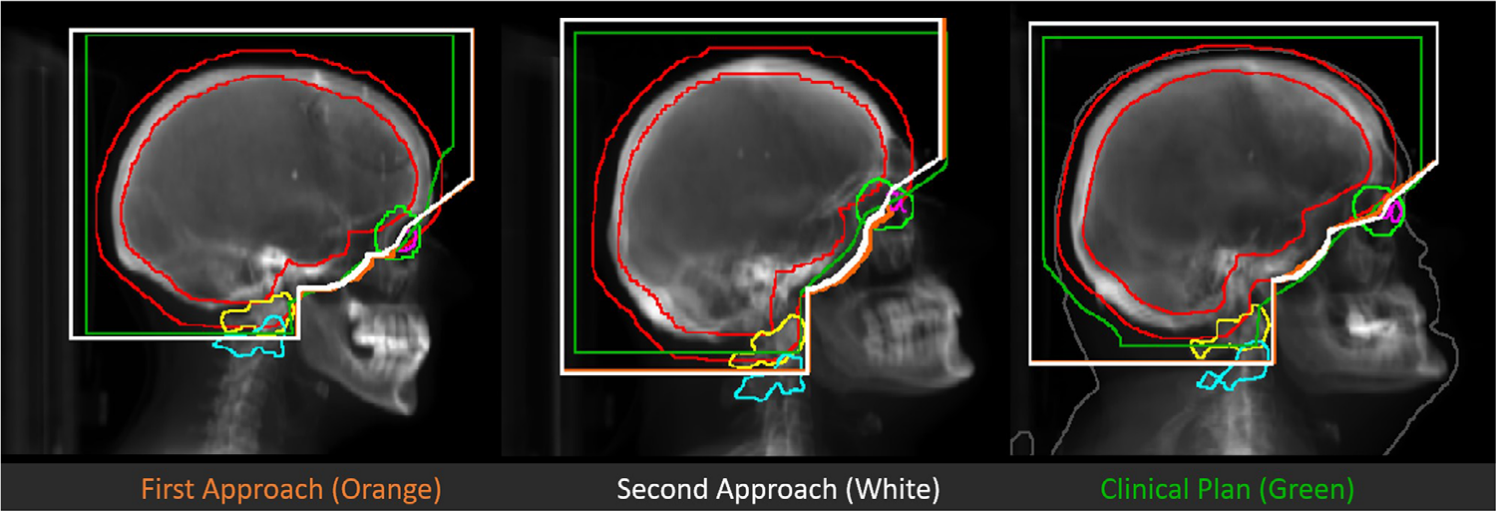
Comparisons of the resulting field apertures from the first (orange) and second (white) approaches and the clinical plans (green). Field apertures in orange and white are almost overlapped.

The field apertures generated using the first and second approaches were compared with each other and with the field apertures in the clinical plans. As shown in Figure 6, the field apertures generated using the first and second approaches were similar and agreed reasonably well with those in the clinical plans, especially for the regions from the eyes to the vertebral bodies.

## 4 DISCUSSION

In this work, we have presented a novel pipeline for automatically generating WBRT treatment field apertures using anatomical landmarks. The pipeline consisted of two approaches to obtaining the anatomical landmarks: The first approach was based on 3D CT segmentation, and the second approach was based on 2D DRR segmentation. We successfully generated field apertures from both approaches, and the generated field apertures were similar to those in the clinical plans and achieved high clinical acceptance rates. We did not include field‐in‐field or skin‐sparing techniques in this pipeline, which remains for further study.

The design of the two parallel field aperture generation approaches could be an effective tool for quality control, as the resulting field apertures were very similar. In practice, if the first approach fails to generate the field apertures, the second approach could be used to check the failure by calculating the distances of the two field apertures. Our future studies will further investigate the criteria for and the effectiveness of identifying automated field aperture check failures following the QA approaches of Rhee et al. and Kisling et al.^10^, ^11^

Field aperture designs vary according to the different purposes for which they are used and preferences in different clinical practices (Figure S2). Our landmark‐based field aperture generation method can be easily configured to suit different clinical purposes and clinical styles. In Figure 2, we detailed how we selected the landmarks to form the boundaries of the field apertures. By adjusting the positions and the shapes of these lines, we allow the flexibility in defining different field aperture shapes to address the needs of different clinical practices. For example, in our hospital, clinicians preferentially use field aperture shapes described as the initial setup (Figure 3, the initial setting), whereas other hospitals may prefer to use a different setting (Figure 3, options 2–6). Our landmark‐based method provides a feasible way of changing the field aperture configurations by introducing user‐specific options.

This technique has not been implemented in the clinic, although we anticipate integrating this technique in the near future (as we have done with other tools^9^). A graphical user interface will be created within RayStation v11.0 (RaySearch, Stockholm, Sweden) to automatically generate a WBRT plan using the treatment planning system's scripting. The user will be asked to input the dose prescription and select settings related to the field aperture design. Once the selection is made, the automation process will begin, including auto‐contouring, field aperture design, dose calculation, and plan generation. A plan report will be generated for clinician review, indicating it is ready for treatment. So that other clinics can benefit from this work, we are also integrating this automated landmark‐based WBRT treatment planning as part of automated radiotherapy planning tools that we aim to make available to clinics with limited resources.^14^ The plan generation process was detailed in Section S1. The automation pipeline will be beneficial for both clinicians and patients, where we can reduce clinicians’ workloads and shorten the treatment planning time down to a few minutes. In the future, we plan to develop a method to predict and automatically adjust specific field shapes by learning from the experience and preferred shapes in clinicians’ routine practices.

## 5 CONCLUSION

In this work, we developed and evaluated a novel pipeline consisting of two landmark‐based field aperture generation approaches for WBRT treatment planning; they are fully automated and customizable. The performance results regarding quantitative and qualitative evaluations demonstrated that the automatically generated plans were comparable with the original clinical plans.

## FUNDING INFORMATION

Wellcome Trust

## CONFLICTS OF INTEREST

The authors declare that there is no conflict of interest that could be perceived as prejudicing the impartiality of the research reported.

## AUTHOR CONTRIBUTIONS

Yao Xiao—writing the manuscript, second approach ROI segmentation deep learning model development, customizable landmark‐based field aperture design and development, and data analysis; Carlos Cardenas—data collection, customizable landmark‐based field aperture design, ROI projection code development, supervising research, manuscript review; Dong Joo Rhee—first approach ROI segmentation deep learning model development, manuscript review; Tucker Netherton—first approach ROI segmentation deep learning model development, manuscript review; Lifei Zhang—DRR generation code development, RPA deployment, manuscript review; Callistus Nguyen—deep learning models deployment, clinical integration, manuscript review; Raphael Douglas – dose data collection, RPA deployment, manuscript review; Raymond Mumme—data collection, manuscript review; Stephen Skett—clinical integration, manuscript review; Tina Patel—clinical integration, manuscript review; Chris Trauernicht—clinical integration, manuscript review; Caroline Chung—clinical review, clinical integration, manuscript review; Hannah Simonds—clinical review, clinical integration, manuscript review; Ajay Aggarwal—clinical review, clinical integration, manuscript review; Laurence Court—writing manuscript, supervising research, manuscript review.

## Supporting information

Supporting Information

Supporting Information

Supporting Information

## Data Availability

The data are not publicly available due to privacy or ethical restrictions.
