# Supplementary figures and images for: Customizable landmark‐based field aperture design for automated whole‐brain radiotherapy treatment planning
Source: J Appl Clin Med Phys. 2022 Nov 22;24(3):e13839. doi: 10.1002/acm2.13839 (PMC10018662; doi:10.1002/acm2.13839)

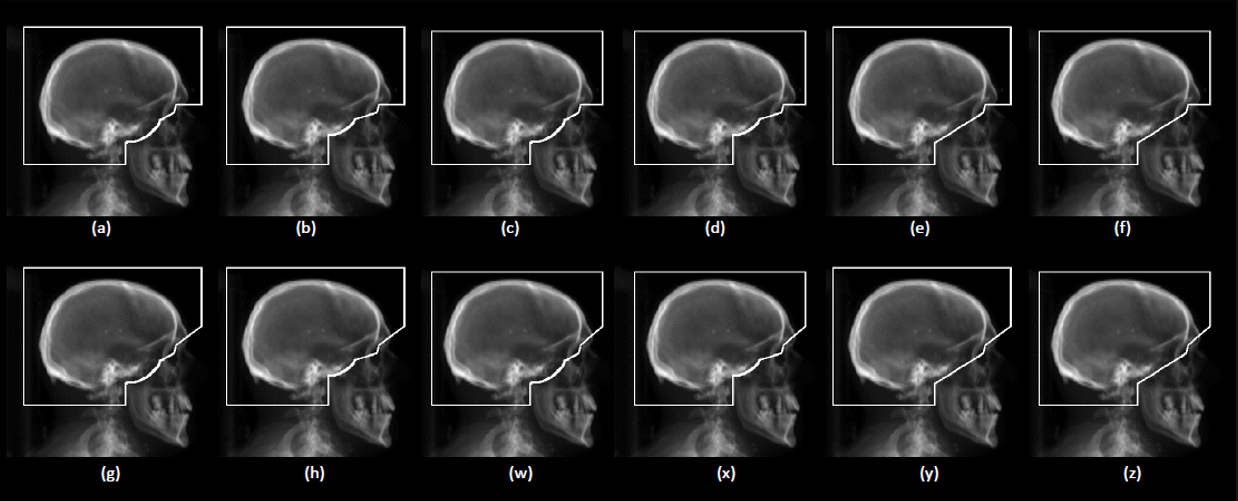

Supplement: Supplementary file 1 — Supporting Information [file ACM2-24-e13839-s003.png]

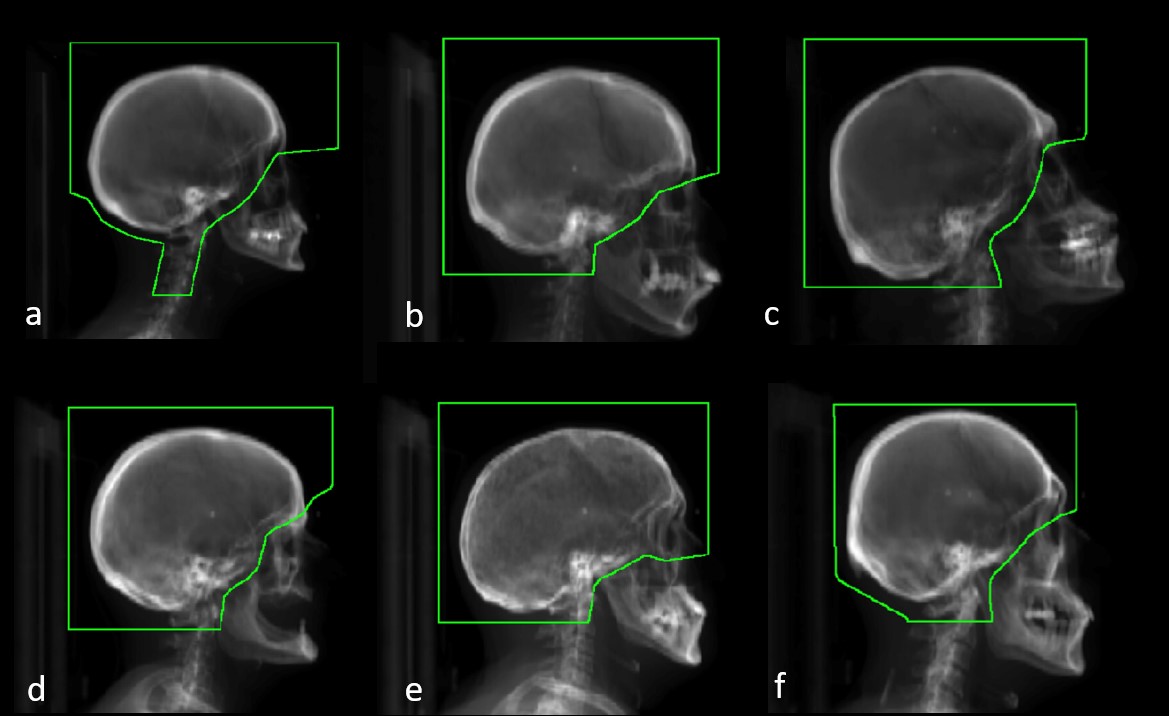

Supplement: Supplementary file 2 — Supporting Information [file ACM2-24-e13839-s001.jpg]
