# Supplementary material for: Customizable landmark‐based field aperture design for automated whole‐brain radiotherapy treatment planning
Source: J Appl Clin Med Phys. 2022 Nov 22;24(3):e13839. doi: 10.1002/acm2.13839 (PMC10018662; doi:10.1002/acm2.13839)
Supplement: Supplementary file 3 — Supporting Information [file ACM2-24-e13839-s002.docx]

Customizable Landmark-Based Field Aperture Design for Automated Whole-Brain Radiotherapy Treatment Planning Supplementary Document

This document contains some additional details and figures not included in the main manuscript.

# S1. Automated Plan Generation

The automatic aperture design algorithms were integrated into the Radiation Planning Assistant (RPA^14^), a web-based tool that is being developed to provide automated contouring and planning solutions to clinics with limited resources. In the RPA, automatically generated apertures are imported into Eclipse treatment planning system (Varian Medical Systems), v15.6, and the Eclipse API (ESAPI) is used to automatically generate the treatment plan.

# S2. Initial Field Aperture Configuration Selection

For each patient, 12 candidate field apertures (Figure S1) were generated based on different combinations of the landmark-based options (Figure 3 options 2, 4, 5, and 6). For example, candidate (a) in Figure S1 was combined using option 2 with a horizontal line, option 4 for along the brain expand, option 5 with a 15 mm brain expansion, and option 6 with a 15 mm skin flash.

**Figure S1.** The 12 candidate field apertures for initial field aperture configuration selection.

# S3. Clinical Variations

Field aperture designs vary according to the different purposes for which they are used and preferences in different clinical practices. Figure S2 provied some field aperture shapes that were used in our clinic, where b) is very close to our initial configuration in Figure 3 1). In addition, the forehead region of a) is similar to option 2) in Figure 2, and e) is similar to option 8) in Figure 2.

**Figure S2.** Clinical variations in the field aperture design.
